# MUC1 promotes cervical squamous cell carcinoma through ERK phosphorylation-mediated regulation of ITGA2/ITGA3

**DOI:** 10.1186/s12885-024-12314-6

**Published:** 2024-05-03

**Authors:** Aiqin Zhao, Yunzhi Pan, Yingyin Gao, Zheng Zhi, Haiying Lu, Bei Dong, Xuan Zhang, Meiying Wu, Fenxia Zhu, Sufang Zhou, Sai Ma

**Affiliations:** 1 Department of Obstetrics and Gynecology, The People’s Hospital of Suzhou New District, Suzhou, 215129 China; 2 Department of Pharmacy, The Affiliated Infectious Diseases Hospital of Soochow University, Suzhou, 215131 China; 3 Affiliated Hospital of Integrated Traditional Chinese and Western Medicine, Nanjing University of Chinese Medicine, Nanjing, 210028 China; 4 Key Laboratory of New Drug Delivery Systems of Chinese Materia Medica, Jiangsu Province Academy of Traditional Chinese Medicine, Nanjing, 210028 China; 5 Department of Pathology, The Affiliated Suzhou Hospital of Nanjing Medical University, Suzhou, 215002 China; 6 Department of Tuberculosis, The Affiliated Infectious Diseases Hospital of Soochow University, Suzhou, 215131 China; 7 Department of Laboratory, The Affiliated Suzhou Hospital of Nanjing Medical University, Suzhou, 215002 China; 8 Gusu School, Nanjing Medical University, Suzhou, 215008 China

**Keywords:** Cervical squamous cell carcinoma, MUC1, ERK, ITGA2, ITGA3

## Abstract

In contrast to the decreasing trends in developed countries, the incidence and mortality rates of cervical squamous cell carcinoma in China have increased significantly. The screening and identification of reliable biomarkers and candidate drug targets for cervical squamous cell carcinoma are urgently needed to improve the survival rate and quality of life of patients. In this study, we demonstrated that the expression of MUC1 was greater in neoplastic tissues than in non-neoplastic tissues of the cervix, and cervical squamous cell carcinoma patients with high MUC1 expression had significantly worse overall survival than did those with low MUC1 expression, indicating its potential for early diagnosis of cervical squamous cell carcinoma. Next, we explored the regulatory mechanism of MUC1 in cervical squamous cell carcinoma. MUC1 could upregulate ITGA2 and ITGA3 expression via ERK phosphorylation, promoting the proliferation and metastasis of cervical cancer cells. Further knockdown of ITGA2 and ITGA3 significantly inhibited the tumorigenesis of cervical cancer cells. Moreover, we designed a combination drug regimen comprising MUC1-siRNA and a novel ERK inhibitor in vivo and found that the combination of these drugs achieved better results in animals with xenografts than did MUC1 alone. Overall, we discovered a novel regulatory pathway, MUC1/ERK/ITGA_2/3_, in cervical squamous cell carcinoma that may serve as a potential biomarker and therapeutic target in the future.

**Supplementary Information:**

The online version contains supplementary material available at 10.1186/s12885-024-12314-6.

## Abstract (plain language summary)

MUC1 is overexpressed in cervical squamous cell carcinoma. MUC1 regulates ERK phosphorylation, and subsequently upregulates ITGA2 and ITGA3 expression to promote tumorigenesis in cervical squamous cell carcinoma. A combination drug regimen targeting MUC1 and ERK achieved better results compared than MUC1 alone.

**Supplementary Information:**

The online version contains supplementary material available at 10.1186/s12885-024-12314-6.

## Introduction

Cervical cancer is the second most common and fourth leading cause of cancer death among females worldwide [1]. Approximately 10% of the global cervical cancer deaths occurred in China due to its large population. Cervical squamous cell carcinoma (CSCC) accounts for 90% of cervical cancer cases [2]. In contrast to the decreasing trends in developed countries, the incidence and mortality rates of CSCC in China have increased significantly since 2000 [3, 4]. Currently, the main early screening method for cervical cancer is the combination of exfoliation cytology and HPV detection, but its sensitivity is not high in clinical diagnosis [5]. More than 50% of patients in China are diagnosed with locally advanced cervical cancer when detected and their treatment response is poor because of the lack of effective treatments [6]. Therefore, screening and identification of innovative prognostic biomarkers and candidate drug targets for CSCC are very urgently needed to improve the survival rate and quality of life of patients.

Mucins, the main components of mucus, are large glycoproteins with high molecular weights. Mucins are essential for various physiological processes, including the protection of epithelial cells and signaling transduction [7–9]. As a membrane-bound mucin, MUC1 is a single-pass transmembrane protein that consists of an extracellular domain, a transmembrane domain, and a cytoplasmic tail [10]. MUC1 is expressed mainly in the epithelial cells of the stomach, intestine and esophagus and acts as a cell surface receptor in normal tissues, participating in signal transmitting and playing important roles in cell adhesion, immune response and other biological functions [11, 12].

Many studies suggest that MUC1 provides a microenvironment conducive to preventing hypoxia, acidity, and other biological conditions that promote cancer progression [13–15]. Overexpression of MUC1 is associated with invasive and metastatic tumors of the colon, pancreas, breast, and oral epithelium [16–19]. In cervical cancer, MUC1 is involved in the malignancy of cervical adenocarcinoma [20]. Patients with cervical adenocarcinoma and high MUC1 expression have a low survival rate and lymph node metastasis [21]. In the squamous type of cervical cancer, MUC1 is more highly expressed in metastatic tumoral cervical tissues than in negative lymph nodes [22, 23]. However, the expression of MUC1 in precancerous lesions of cervical squamous cell carcinoma remains unclear. Therefore, the implications and regulatory mechanism of MUC1 in cervical cancer need further exploration.

In this study, we explored the expression of MUC1 in cervical squamous cell carcinoma. We found that MUC1 was overexpressed in CSCC tissues. The expression of MUC1 was greater in neoplastic tissues than in non-neoplastic tissues of the cervix. As the severity of cervical precancerous lesions increased, the expression of MUC1 also increased. Then, we revealed the potential role and regulatory mechanism of MUC1 in cervical squamous cell carcinoma. MUC1 overexpression promoted the proliferation and invasion of CSCC cells via ERK phosphorylation, which upregulated ITGA2 and ITGA3 expression. Targeting the MUC1-ERK axis could significantly suppress tumors in vivo. Taken together, the present study demonstrated a novel regulatory MUC1/ERK/ITGA_2/3_ pathway and MUC1 may be used as a potential biomarker for early detection of cancer and therapeutic target in CSCC.

## Materials and methods

### Tissue specimens and immunohistochemical staining

Fresh cervical cancer tissues and adjacent tissues were obtained from surgical resection specimens collected by the Department of Obstetrics and Gynecology at the People`s Hospital of SND, Jiangsu Province, China. None of the patients received treatment prior to surgery, and all patients signed informed consent forms for tissue sampling and the isolation and storage of DNA, RNA and protein. The study was approved by the Ethics Committee of the People`s Hospital of SND (2018-013). Tumor and normal operative margin tissues from the same patients were separated by experienced pathologists, and immediately stored at -80℃ until use, as described previously [24].

As described previously, paraffin-embedded tissue was prepared for IHC [25]. The sections were incubated with an anti-MUC1 antibody (sc-7313; Santa Cruz Bio, USA; 1:100) overnight at 4 °C and then treated according to the PV-9000 kit (3550; ZSbio, China) and DAB kit (3502; ZSbio). The staining intensity of MUC1 was scored into four grades: 0 (negative), 1 (weak) as low expression, 2 (moderate) and 3 (strong) as high expression.

### Cell culture and treatments

The human cervical squamous cell carcinoma lines SW756, SiHa, CaSki, C33A and ME180 were purchased from the Chinese Academy of Sciences Cell Bank (Shanghai, China) [26]. SW756, SiHa and CaSki cells were cultured in RPMI 1640 medium (11875093; Gibco, USA) supplemented with 10% (vol/vol) fetal bovine serum (10099158; Thermo Scientific, USA), penicillin (15140148; 100 U/mL, Gibco) and streptomycin (15140148; 100 mg/mL, Gibco) at 37 °C in a humidified incubator containing 5% CO_2_. SW756 and ME180 cells were cultured in Dulbecco’s modified Eagle’s medium (11965092; Gibco, USA) with 10% (vol/vol) fetal bovine serum, penicillin and streptomycin at 37℃ in a humidified incubator containing 5% CO_2_. All of the cell lines were tested for Mycoplasma and verified by us using short tandem repeat analysis.

The sgRNAs against human MUC1, small interfering RNAs against human ITGA2, ITGA3 and negative controls were synthesized by GenePharma (China, Supplementary Table 1). As described above, sgRNAs and siRNAs were transfected into cells using Lipofectamine 2000 (11668500; Life Technologies, USA) according to the manufacturer’s instructions. Then, cells stably transfected with the sgRNAs were selected with puromycin (A1113803; Gibco; 1 µg/mL) [24].

### Cell matrigel invasion assays

The migration and invasion assays were performed on Transwell plates (3422, 8 μm; Corning Costar, USA) as described previously [27]. For the cell migration assay, 1 × 10^5^ SiHa cells or 1 × 10^5^ CaSki cells were seeded on a fibronectin-coated polycarbonate membrane insert in a Transwell apparatus. RPMI 1640 containing 20% FBS was added to the lower chamber. After incubation for 24 h at 37 °C, the cells on the top surface of the insert were removed with cotton swabs. Then, the cells were fixed with 100% methanol, stained with 0.5% crystal violet (61135; Sigma-Aldrich, USA) and imaged via microscopy. For the invasion assay, the procedure was similar to that for the migration assay, except the Transwell membrane was precoated with 300 ng/mL Matrigel (356255; Corning), and the cells were incubated for 48 h at 37 °C.

### Cell proliferation assay

The cell proliferation assay was performed as described previously [24]. A total of 1 × 10^3^ SiHa or CaSki cells were seeded on 96-well plates with 3 replicates. Viable cells were quantified every two days by using 3-(4,5-dimethylthiazol-2-yl)-2,5-diphenyltetrazolium bromide (475989; MTT, Sigma-Aldrich, USA). The absorbance at 570 nm was measured using a Thermo Varioskan LUX Reader (Thermo Fisher, USA).

### Colony formation assay

The colony formation assay was performed as described previously [24]. Briefly, 1 × 10^3^ SiHa or CaSki cells were plated in each well of a 24-well plate (3524; Corning) with 3 replicates. After culture for 7 days, the cells were washed with PBS and fixed with 100% methanol for 1 h, stained with 0.5% crystal violet and imaged via light microscopy (Leica, Germany) to determine the number of cells.

### Antibodies and western blot analysis

Total protein was isolated from cells by using lysis buffer (P0013B; Beyotime, China). For high-throughput protein detection, we cut the membrane corresponding to the protein molecular weight prior to incubation with antibodies. Immunoblotting was performed with primary antibodies against MUC1 (sc-7313; Santa Cruz, USA; 1:200), ITGA2 (ab133557; Abcam, USA; 1:500), ITGA3 (ab131055; Abcam, USA; 1:500), ERK1/2 (4695; CST, USA; 1:1000), phosphorylated ERK1/2 (Thr202/Tyr204) (4370; CST, USA; 1:500) and GAPDH (60004-1-Ig; Proteintech, China; 1:5000) as the control and followed by the secondary antibodies (CW0102S and CW0103S; CWbiotech, China; 1:5000). The specific bands were visualized with super enhanced chemiluminescence (ECL) detection reagent (180–5001; Tanon, China).

### RNA isolation and real-time PCR

Total RNA was isolated from cells using the E.Z.N.A.Total RNA Kit II (R6934; Omega BioTek, USA) as described previously [27]. The isolated RNA was used as template for reverse transcription reactions with PrimeScript™ RT Master Mix Kit (RR036B; Takara, Japan).

Quantitative real-time PCR analysis was performed using SYBR Green Real-Time PCR Master Mixes (4309155; Thermo Fisher) on a LightCycler 480 II Real-Time PCR Detection System (Thermo Fisher). The relative mRNA levels of the target genes were normalized to that of the endogenous reference GAPDH. The primers used are listed in Supplementary Table 2.

### Animal experiments

The Committee on the Use of Live Animals of Soochow University approved all the animal experiments, and all methods are reported in accordance with ARRIVE guidelines [28]. 10 six-week-old female nude mice purchased from ZiYuan Co.Ltd (Hangzhou, China) were subcutaneously injected with 1 × 10^6^ CaSki cells harboring gMUC1-1 (*n* = 5 per group) or the negative control to evaluate tumor growth, as described previously [24, 25]. The mice were sacrificed after 40 days. For the tumor metastasis assay, 12 six-week-old female NOD/SCID mice were injected with 1 × 10^6^ CaSki cells harboring gMUC1-1 or the negative control via the tail vein (*n* = 6 per group). The mice were sacrificed after 6 weeks. Metastastic nodules in lung tissues were fixed in Bouin’s solution (PH0976, Phygene, China), and the number of metastases was determined. The tumor samples were embedded in paraffin, cut into 5-µm sections and stained with hematoxylin and eosin (H&E). For inhibition of tumor growth, 15 six-week-old female nude mice were subcutaneously implanted with 1 × 10^6^ CaSki cells. After one week, the mice were equally divided into three groups according to the mean tumor volume (*n* = 5 per group). Mice were injected intraperitoneally with PBS and tail vein injection of non-silencing siRNA incubated with Entranster-in vivo (18668-11-1; Engreen Biosystem, China; three times per week, vehicle control). Mice were also injected via the tail vein with MUC1 siRNA only (and intraperitoneally injection with PBS) or in combination of intraperitoneally injection with drug MK-8353 (MK-8353; MCE, USA; 10 mg/kg; three times per week) in PBS and tail vein injection of MUC1 siRNA incubated with Entranster-in vivo (Engreen Biosystem; three times per week). Tumor size (length × width^2^ × 0.52) was measured, and the mice were sacrificed at the indicated time points. The weights of the xenografts were measured, and the xenografts were imaged.

### RNA-seq analysis

As described previously, the total amount and integrity of the RNA were assessed using an RNApure Tissue and Cell Kit (CW0560S; CWBIO), and the RNA was sequenced via the 150 bp paired method [27]. The HISAT2 (2.2.1, https://daehwankimlab.github.io/hisat2/), SAMtools (1.19, https://www.htslib.org/), and HTseq-count (2.0.3, https://htseq.readthedocs.io/en/release_0.11.1/count.html) packages were used to align 150 bp paired-end reads with hg19 (UCSC). The expression of genes and transcripts was quantified using Cufflinks tools (2.2.1, http://cole-trapnell-lab.github.io/cufflinks/).

### Statistical analysis

All the statistical analyses were performed using IBM SPSS Statistics 22.0 (IBM, USA). The experimental results were statistically evaluated using Student’s t test for two groups and ANOVA for three or more groups. Fisher’s least significant difference (LSD) test was used for further analysis under the significant result of ANOVA. Log-rank (Mantel-Cox) was used to access the statistically significance of the difference in Kaplan-Meier (KM) analysis. *P* < 0.05 was considered to indicated statistical significance.

## Results

### MUC1 is highly expressed in human cervical squamous cell carcinoma tissues

**Fig. 1:**
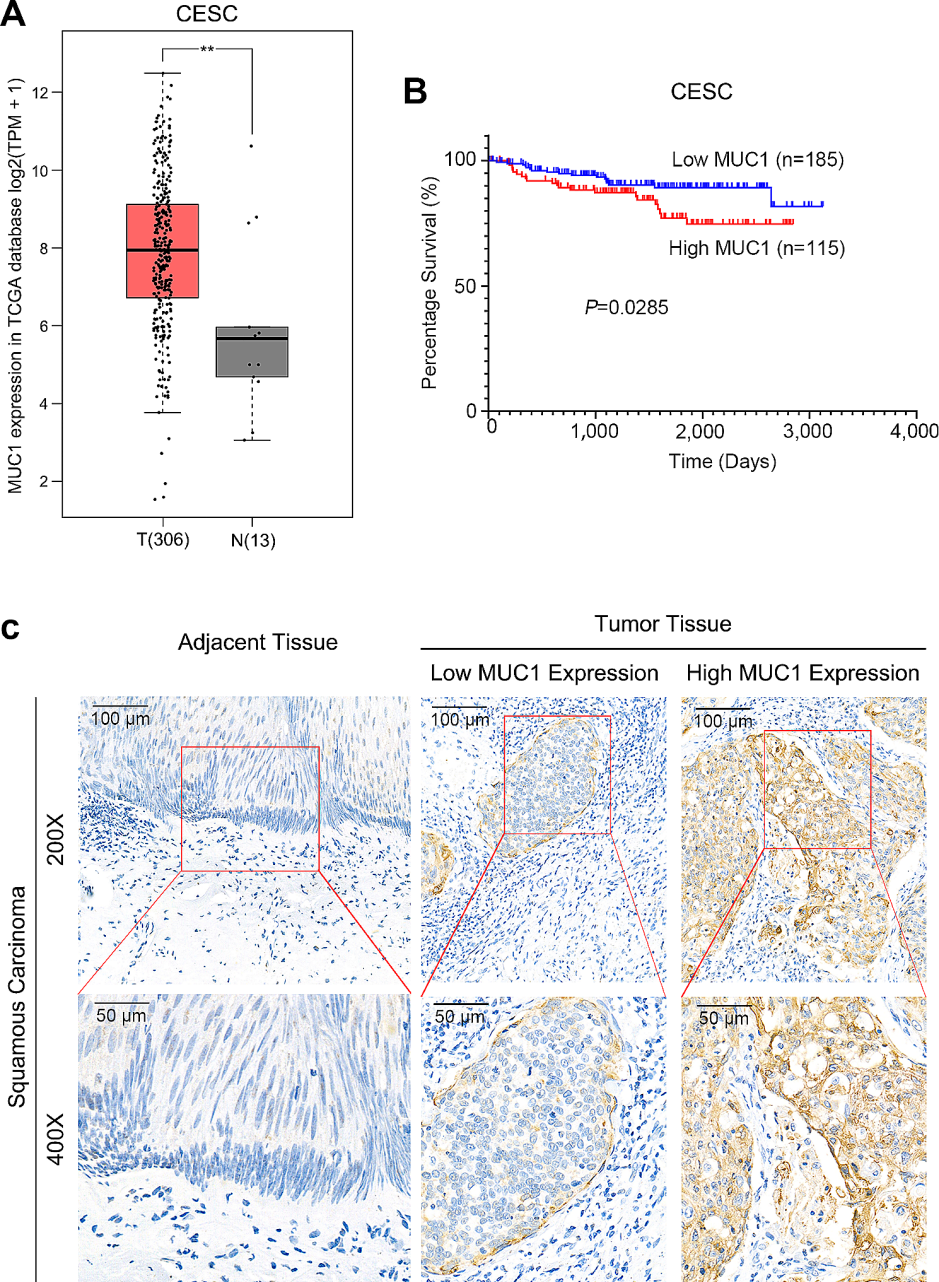
MUC1 is highly expressed in human cervical squamous cell carcinoma tissues. (**A**) RNA-seq expression of MUC1 in the TCGA CESC cohort. ANOVA was used to access the statistically significance of the difference (**B**) Kaplan-Meier (KM) analysis of 300 cases in the GSE44001 cohort survival curves between the lower MUC1 expression and higher MUC1 expression groups. Log-rank (Mantel-Cox) was used to access the statistically significance of the difference (**C**) Representative immunohistochemical images of MUC1 expression in tumor and adjacent nontumor normal epithelial tissues *, *p* < 0.05; **, *p* < 0.01

According to the Gene Expression Profiling Interactive Analysis (GEPIA) database (http://gepia.cancer-pku.cn/), MUC1 was highly expressed in cervical squamous cell carcinoma (Fig. 1A) [29]. Kaplan–Meier analysis of 300 cases in the GSE44001 cohort revealed that patients with high MUC1 expression had significantly poorer overall survival than patients with low MUC1 expression (*P* = 0.0285; Fig. 1B). We further performed immunohistochemistry (IHC) analysis on our tissue arrays of 40 paraffin-embedded adjacent sections of normal cervical tissue and CSCC tissue. Tumors that displayed moderate or strong immunostaining were classified as having high MUC1 expression, and the remainder of the tumors that displayed negative or weak immunostaining were classified as having low MUC1 expression. In the 40 CSCC samples, high MUC1 expression was observed in 72.5% (29/40) of the tumors and 32.5% (13/40) of the adjacent tissues, and the difference between tumors and normal tissues was significant (*P* < 0.001; Fig. 1C and Supplementary Table 3).

### MUC1 expression gradually increased with the progression of cervical lesions

**Fig. 2:**
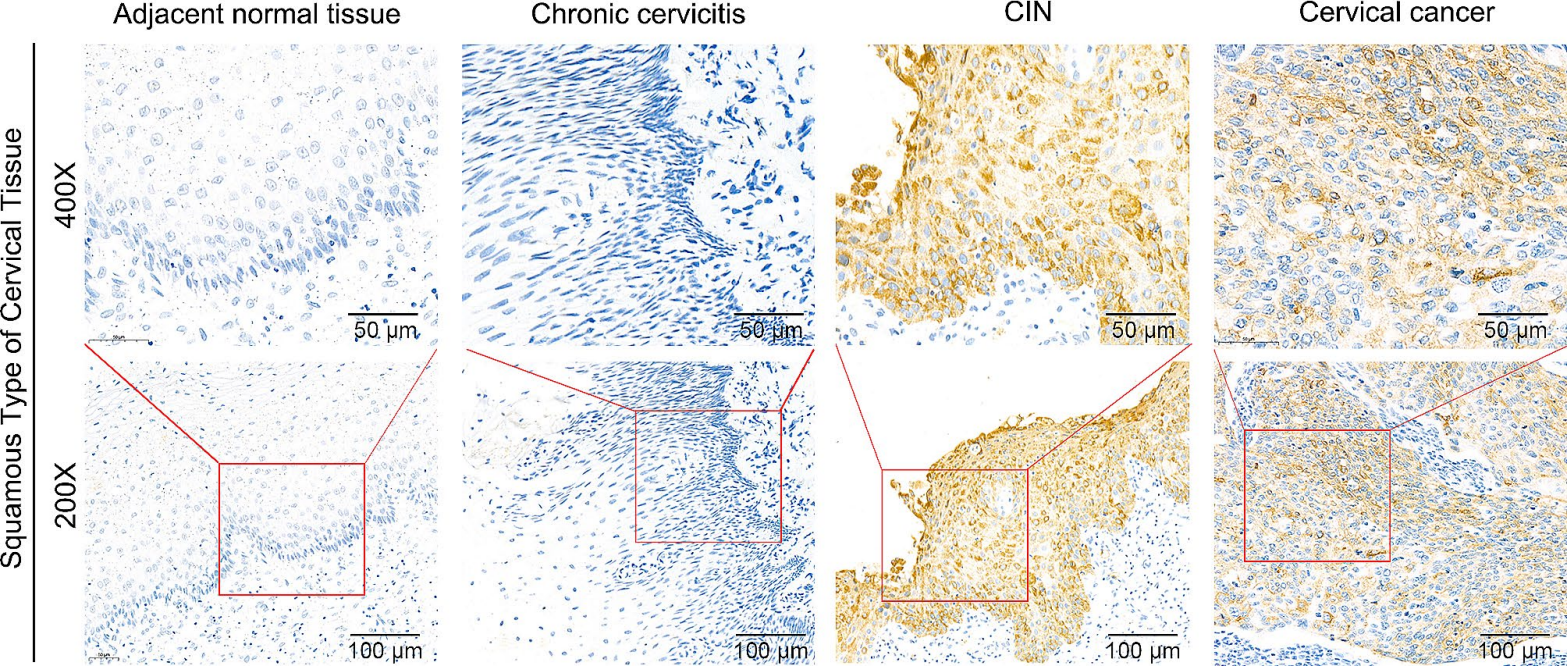
MUC1 expression gradually increases with the progression of cervical lesions. Representative immunohistochemical images of MUC1 expression in adjacent normal tissues, chronic cervicitis, cervical intraepithelial neoplasia (CIN) tissues and cervical squamous cell carcinoma (CSCC) tissues

To explore the role of MUC1 in the early diagnosis of cervical squamous cell carcinoma, we analyzed MUC1 expression in different types of cervical neoplasms. 10 of the 25 chronic cervicitis tissues were shown to have high MUC1 expression (40.0%, Fig. 2, Supplementary Table 4). More than half of the cervical intraepithelial neoplasm (CIN) tissues exhibited MUC1 expression (30/51, 58.8%, Fig. 2, Supplementary Table 4). As mentioned above, 72.5% of cervical cancers strongly expressed of MUC1. These results indicated that the expression of MUC1 increased with the degree of cervical neoplasm, and the difference was significant (*P* = 0.034, Supplementary Table 4).

### Loss of MUC1 alleviates the tumorigenic ability of cervical squamous cell carcinoma cells

**Fig. 3:**
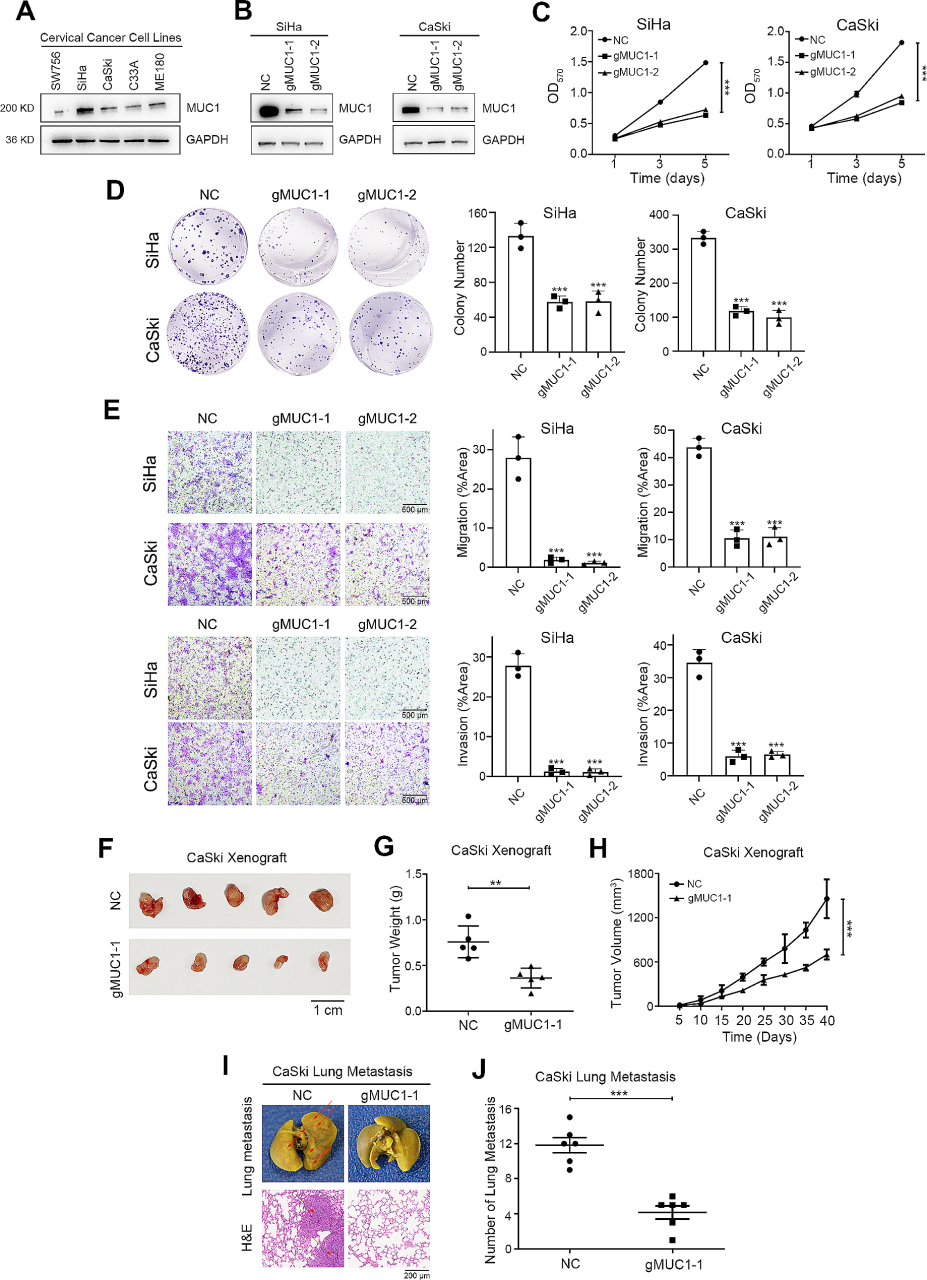
Loss of MUC1 alleviates the tumorigenic ability of cervical squamous cell carcinoma cells. (**A**) Western blot analysis of MUC1 expression in CSCC cell lines. For high-throughput protein detection, we cut the membrane corresponding to the protein molecular weight prior to incubation with antibodies (**B**) Western blot analysis of MUC1 expression in SiHa and CaSki cells after sgRNA transfection. For high-throughput protein detection, we cut the membrane corresponding to the protein molecular weight prior to incubation with antibodies (**C**) Cell proliferation assay after MUC1 knockout in SiHa and CaSki cells, *n* = 3 (**D**) Representative images and statistical plots of colony growth in SiHa and CaSki cells after MUC1 knockout, *n* = 3 (**E**) Representative images and statistical plots of invasion assays in SiHa and CaSki cells after MUC1 knockout, *n* = 3 (**F**) Representative images of tumors from the indicated groups, *n* = 5 (**G**) The weights of tumors from the indicated groups, *n* = 5 (**H**) Tumor growth curves of tumors from the indicated groups, *n* = 5 (**I**) Representative photos of fixed lung tissues and the results of H&E staining. The arrows indicate the lung metastatic nodules, *n* = 6 (**J**) The number of metastatic nodules from the indicated groups, *n* = 6 The mean ± SEM are shown. **, *p* < 0.01; ***, *p* < 0.001

First, we detected the expression of MUC1 in five CSCC cell lines by Western blot analysis and found that MUC1 was strongly expressed in highly malignant SiHa and CaSki cells but rarely expressed in less malignant SW756 cells (Fig. 3A and Supplementary Fig. 1A). CRISPR was used to knock out MUC1 to assess the biological role of MUC1 in CSCC cells (Fig. 3B and Supplementary Fig. 1B). We found that the proliferation of CSCC cells decreased after MUC1 knockout (Fig. 3C). The colony-forming capacity of the cells was also inhibited after elimination of MUC1 (Fig. 3D). Then we investigated the role of MUC1 in regulating the migration and invasion of CSCC cells. Transwell assays showed that MUC1 knockout significantly decreased the migration and invasion of cervical cancer cells (Fig. 3E).

A series of in vivo experiments were also performed. MUC1 knockout and control cells of CaSki cells were injected subcutaneously into the underarms of nude mice. The results showed that the xenograft size of the MUC1-KO cells was significantly lower than that of the negative control cells (Fig. 3F and H). Moreover, we performed a pulmonary metastasis assay to assess the effect of MUC1 on metastasis. CaSki MUC1-KO and control cells were injected via the tail vein into NOD/SCID mice. Cancer cells in the MUC1-KO group lost the ability to metastasize to the lung. H&E staining of paraffin-embedded lung tissues further confirmed these findings (Fig. 3I and J). Collectively, these data demonstrated that MUC1 knockout alleviated the proliferation and invasion of CSCC cells.

### High MUC1 expression accelerates tumor development via ERK phosphorylation and ITGA2/3

**Fig. 4:**
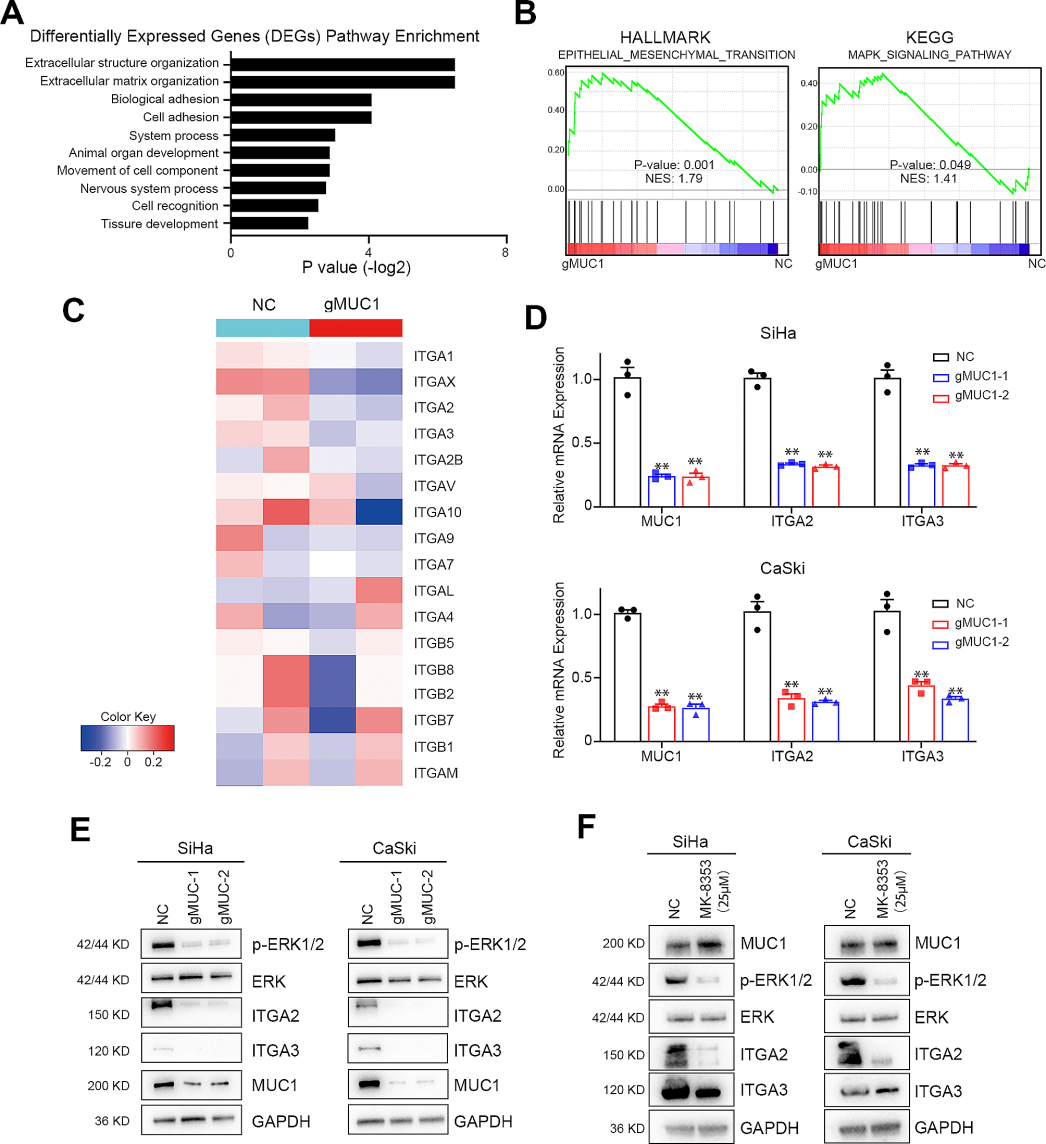
High MUC1 expression accelerates tumor development via ERK phosphorylation and ITGA2/3. (**A**) Top 10 enriched pathways of differentially expressed genes following MUC1 elimination in CaSki cells (**B**) GSEA of RNA-seq data from negative control and MUC1 knockout in CaSki cells (**C**) Heatmap of the variation in the ITGA and ITGB families upon MUC1 knockout in CaSki cells (**D**) Real-time analysis of MUC1, ITGA2 and ITGA3 mRNA expression in negative control and MUC1 knockout SiHa and CaSki cells, *n* = 3 (**E**) Western blot analysis of p-ERK, ERK, ITGA2, ITGA3 and GAPDH in negative control and MUC1 knockout SiHa and CaSki cells. For high-throughput protein detection, we cut the membrane corresponding to the protein molecular weight prior to incubation with antibodies (**F**) Western blot analysis of MUC1, p-ERK, ERK, ITGA2, ITGA3 and GAPDH in SiHa and CaSki cells treated with DMSO or the ERK inhibitor MK-8353. For high-throughput protein detection, we cut the membrane corresponding to the protein molecular weight prior to incubation with antibodies The means ± SEM are shown. **, *p* < 0.01

To clarify the mechanism by which MUC1 enhances the proliferation and metastasis of CSCC cells, we compared the changes in RNA levels between the MUC1 knockout group and the control group by RNA-seq. After MUC1 knockout, the extracellular matrix and cell adhesion signaling pathways were significantly enriched (Fig. 4A and Supplementary Table 5). The DEGs that were differentially expressed after MUC1 KO were subsequently subjected to unbiased Gene Ontology (GO) analysis and gene set enrichment analysis (GSEA). The extracellular matrix organization- and cell adhesion-related pathways were the most common according to the GO analysis. Furthermore, the epithelial-mesenchymal transition (EMT) pathway was significantly enriched according to hallmark analysis, and the MAPK signaling pathway was strongly enriched according to KEGG analysis. The results of bioinformatics analysis indicated that MUC1 expression affected the regulation of the extracellular matrix through the EMT pathway and MAPK pathway (Fig. 4B). In detail, we found that several members of the ITGA and ITGB superfamilies were enriched according to the GO and GSEA results. Therefore, we focused on the analysis of the expression levels of these genes (Fig. 4C). The data suggest that ITGA1, ITGA2, ITGA3 and ITGAX are related to MUC1 expression. Real-time quantitative PCR confirmed that the expression of ITGA2 and ITGA3 was decreased at the mRNA level (Fig. 4D). Further Western blot assays showed that ITGA2 and ITGA3 were inhibited when MUC1 was knockout (Fig. 4E).

Furthermore, we demonstrated that MAPK pathway was activation by MUC1 through the suppression of ERK phosphorylation and the stabilization of total ERK after MUC1 knockdown (Fig. 4E and Supplementary Fig. 2). We subsequently used a novel ERK inhibitor with clinical potential, MK-8353, to restrain the activation of ERK phosphorylation [30]. The interaction between ITGA2 and ITGA3 was abolished after ERK phosphorylation was inhibited (Fig. 4F and Supplementary Fig. 3). Overall, we identified a novel pathway, MUC1/ERK/ITGAs, in cervical squamous cell carcinoma.

### ITGA2 promote the tumorigenic ability of cervical squamous cell carcinoma cells

**Fig. 5:**
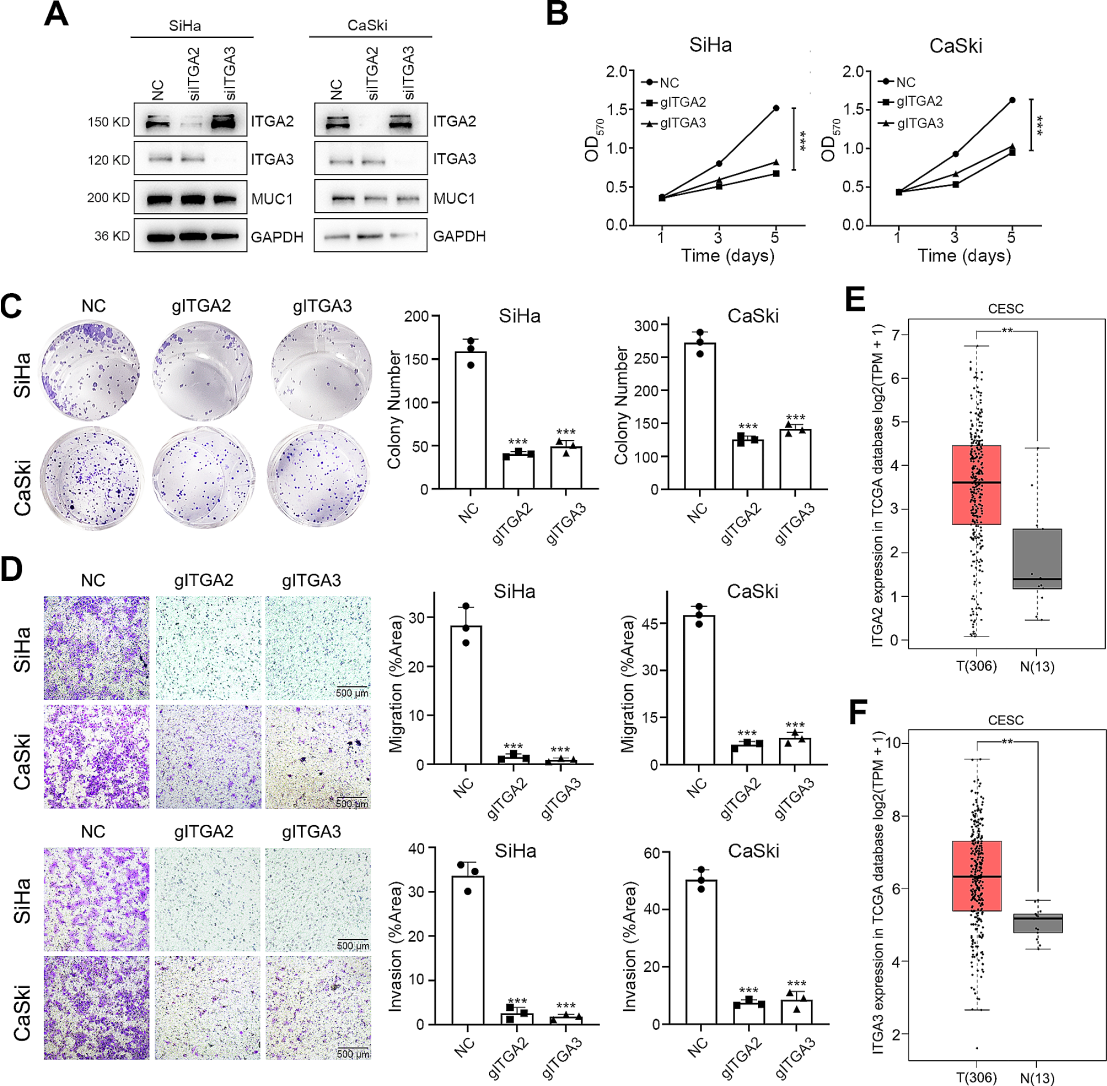
ITGA2 promote the tumorigenic ability of cervical squamous cell carcinoma cells. (**A**) Western blot analysis of ITGA2, ITGA3, MUC1 and GAPHD expression in SiHa and CaSki cells after ITGA2 or ITGA3 knockdown. For high-throughput protein detection, we cut the membrane corresponding to the protein molecular weight prior to incubation with antibodies (**B**) Cell proliferation assay after MUC1 knockout in SiHa and CaSki cells, *n* = 3 (**C**) Representative images and statistical plots of colony growth in SiHa and CaSki cells after MUC1 knockout, *n* = 3 (**D**) Representative images and statistical plots of invasion assays of SiHa and CaSki cells after MUC1 knockout, *n* = 3 (**E**) The RNA-seq expression of ITGA2 in the TCGA CESC cohort. ANOVA was used to access the statistically significance of the difference (**F**) The RNA-seq expression of ITGA3 in the TCGA CESC cohort. ANOVA was used to access the statistically significance of the difference The mean ± SEM are shown. **, *p* < 0.01; ***, *p* < 0.001

We subsequently explored the role of ITGA2 and ITGA3 in CSCC cell lines. Small interfering RNAs (siRNAs) were used to alter ITGA2 and ITGA3 expression. The expression of MUC1 was stable after ITGA2 or ITGA3 knockdown, confirming that ITGA2 and ITGA3 are downstream molecules of MUC1 (Fig. 5A and Supplementary Fig. 4). The proliferation of CSCC cells decreased after ITGA2 and ITGA3 inhibition (Fig. 5B). We found that colony-forming capacity was also inhibited after knockdown of ITGA2 and ITGA3 (Fig. 5C). Transwell migration and invasion assays showed that reducing ITGA2 and ITGA3 expression significantly decreased the migration and invasion of cervical cancer cells (Fig. 5D). Moreover, according to the GEPIA database, ITGA2 and ITGA3 were highly expressed in CSCC tissues (Fig. 5E and F). Collectively, these data demonstrated that the ITGA family of ITGA2 and ITGA3 are novel oncogenes in CSCC and may be new therapeutic targets for cervical squamous cell carcinoma.

### Blockade of the MUC1/ERK pathway suppresses cervical squamous cell carcinoma growth

**Fig. 6:**
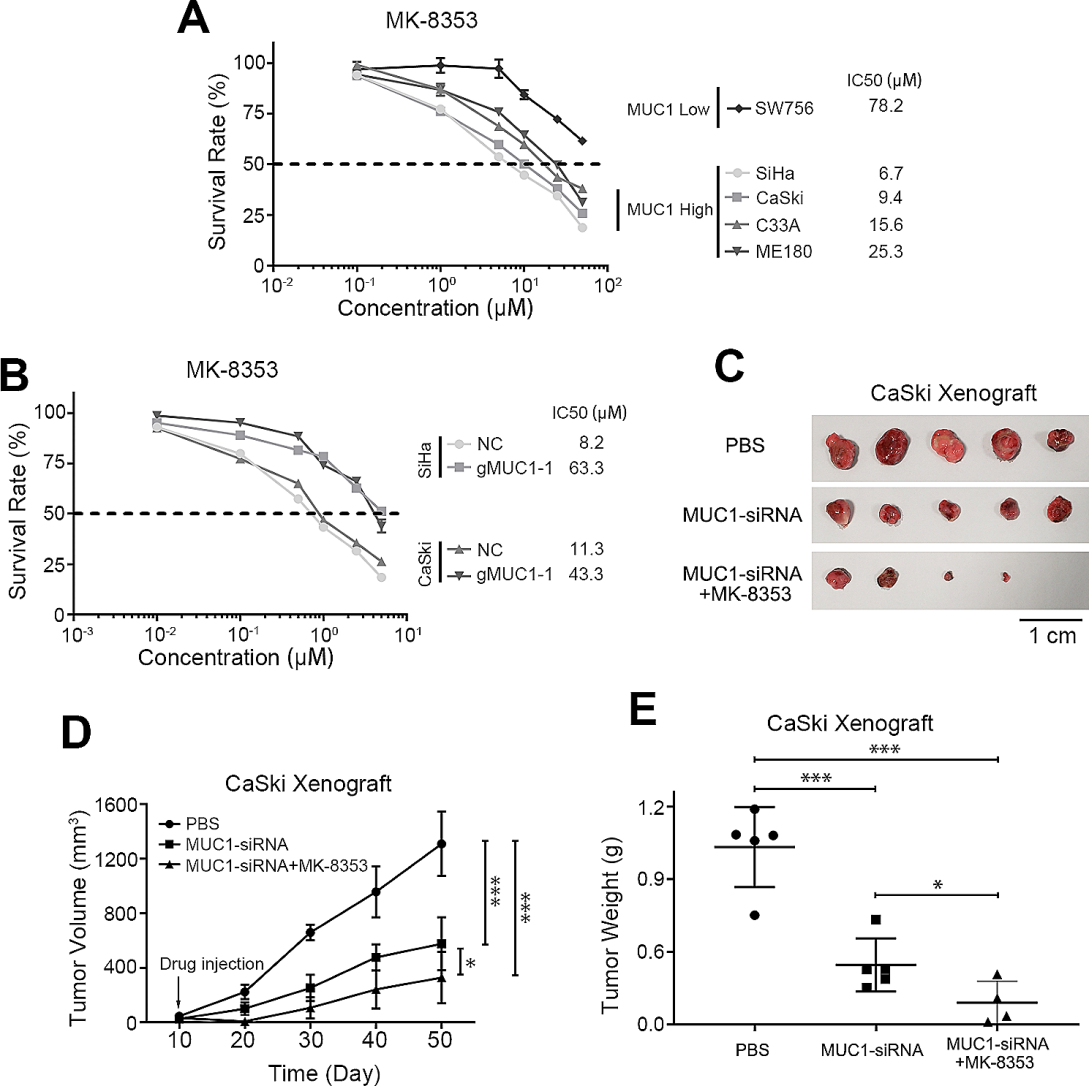
Blockade of the MUC1/ERK pathway suppresses cervical squamous cell carcinoma growth. (**A**) Short-term cell viability assay for determining the IC50s of MK-8353 in different CSCC cell lines, *n* = 5 (**B**) Survival rate of MUC1 wild-type cells and MUC1-KO cells (gMUC1-1) treated with different concentrations of MK-8353, *n* = 5 (**C**) Representative images of tumors from the indicated groups are shown, *n* = 5 (**D**) The weights of tumors from the indicated groups, *n* = 5 (**E**) Tumor growth curves of tumors from the indicated groups, *n* = 5 The means ± SEM are shown. *, *p* < 0.05; ***, *p* < 0.001

In the present study, we revealed that MUC1 effectively induced the proliferation and metastasis of cancer cells via the MUC1-ERK pathway. Overexpression of MUC1 and phosphorylation of ERK play key roles in the progression of CSCC. Based on these findings, we explored the therapeutic potential of this approach in cervical cancer by targeting this pathway. We treated different cervical cells with MK-8353 and found that the IC50 value was dependent on the relative expression of MUC1 (Fig. 6A). The CSCC cell lines SiHa and CaSki exhibited high sensitivity to MK-8535 because of the high expression of MUC1, but strong tolerance to the inhibitor was observed in SW756 cells with low MUC1 expression. When MUC1 was by knocking out its expression, the inhibitory effect of MK-8353 on CSCC cells was significantly reduced (Fig. 6B). Taken together, these results indicated that the inhibitory effect of MK-8353 depended on the expression of MUC1 in cervical squamous cell carcinoma.

Given the above results, we designed a drug combination therapy to target the MUC1/ERK pathway to evaluate the potential antitumor therapeutic effect *in vivo.* In addition to MUC1 siRNA-mediated inhibition of MUC1 expression, MK-8353 was used to block the phosphorylation of ERK. We subcutaneously injected 1 × 10^6^ CaSki cells into the axilla of nude mice. After the tumor xenografts reached a volume of approximately 100 mm^3^, the mice were divided into three different experimental groups. One group served as the control and received non-silencing siRNA and PBS, while the other two groups received MUC1-siRNA; additionally, one of these MUC1-siRNA groups was treated with MK-8353, a specific inhibitor of the ERK pathway. The combination of MUC1 suppression and ERK inhibition had a more pronounced effect on suppressing the growth of the xenografts than MUC1 suppression alone (Fig. 6C-E and Supplementary Fig. 5). These findings suggests that the MUC1/ERK pathway is an important driver in the growth and progression of cervical squamous cell carcinoma.

## Discussion

Herein, we investigated the mechanism of MUC1 and identified a novel regulatory pathway in cervical squamous cell carcinoma. MUC1 was overexpressed in CSCC, activating the phosphorylation of ERK, which enhanced the expression of ITGA2 and ITGA3, thereby promoting the malignant progression of cervical cancer cells.

Early cancer detection is a challenge in general clinical practice. In cervical cancer, cervical intraepithelial neoplasia (CIN) is the abnormal growth of cells on the surface of the cervix that can potentially lead to cancer. In general, CIN arises from a population of squamocolumnar junctional cells or from metaplastic epithelial cells [31]. Persistent infection with high-risk HPV is closely related to cervical intraepithelial neoplasia [32]. Studies have shown that persistent microbial infections induce PD-L1 expression on macrophages, leading to T cell inactivation and accumulation of tumor-promoting cytokines [33]. IL-11 is a tumor-promoting cytokine and is involved in the proliferation and migration of precancerous cells through the NF-κΒ signal transduction [34]. Tumor-promoting cytokines such as IL-11 also induce differentiation of immunosuppression cells through activation of STAT3 signaling pathway [35]. Thus, the combination of HPV tests and cervical cytology is a commonly accepted method for the early diagnosis of CIN and cervical cancer. However, distinguishing benign from malignant early cervical lesions is difficult due to morphological overlap and interobserver variability [36, 37]. In addition, p16 positivity and human papillomavirus (HPV) detection are not useful for identifying lesions that may progress into cancer [38]. This phenomenon is because persistent infection with HPV is necessary, but not sufficient for the development of cervical cancer [39]. Therefore, clarifying the mechanism during the pathogenic infection and identifying markers that are helpful for the early detection of CIN with an increased risk of progression is highly important [40].

Mucins have been explored as biomarkers for early cancer detection and disease prognosis [41]. CA125, also called MUC16, is one of the best-known biomarkers used for the diagnosis of ovarian cancer and is currently being investigated for other malignancies. Other mucins, such as MUC1, MUC2 and MUC5AC, are rarely expressed in normal tissues but are expressed in the early stages of lung cancer, breast cancer, and colon cancer [42, 43]. In cervical cancer, MUC1, MUC4 and MUC20 were reported to be overexpressed in the squamous type [44]. In this study, we further analyzed the expression of the MUC1 protein by immunohistochemical staining of cervical lesions at different stages, from chronic cervicitis to CIN to cervical squamous cell carcinoma to explore the potential of MUC1 in the early diagnosis of cervical squamous cell carcinoma. We found that the expression of MUC1 increased from non-neoplastic glandular lesions of the cervix to cervical cancer, which was similar to the findings of other studies [23]. These results suggest that the detection of MUC1 expression may serve as an early prediction method for precancerous lesion progression to CSCC.

However, the function and regulatory mechanism of MUC1 in cervical cancer has rarely been studied. In the present study, we found that knockout of MUC1 decreased the tumorigenic ability of CSCC cells. The proliferation and invasion of cancer cells were inhibited after MUC1 was eliminated, which was similar to the findings of studies of many other tumors [45, 46]. By RNA-seq, we found that MUC1 overexpression was associated with the extracellular matrix and cell adhesion pathways. In particular, GSEA revealed that MUC1 knockout resulted in significant inhibition of MAPK pathway-related genes. Further Western blot analysis indicated that MUC1 was required for ERK phosphorylation in cervical cancer cells. ERK belongs to the MAPK family, which transfers extracellular signals to intracellular target molecules and plays a key role in signal cascade. ERK is abnormally high expressed in the tissues of persistent microbial infections and could promote the transformation of precancers cells into tumor cells [47]. MUC1-C could induce ERK phosphorylation in breast cancer [48]. MUC1 is also required for MAPK activation and self-renewal in acquired Osimertinib-resistant NSCLC [49]. This regulatory effect may be due to MUC1 being a transmembrane mucin that consists of an N-terminal extracellular domain (MUC1-N) and a C-terminal intracellular domain (MUC1-C) and expression at the apical surface of epithelial cells. Enzymes such as ADAM17 (a disintegrin and metalloprotease domain containing protein-17) or matrix metalloproteases (MMPs) can cleave MUC1-N from the cell surface, leaving MUC1-C behind as a putative receptor on the cell surface [50, 51]. MUC1-C contains various post-translational modifications. These residues act as binding sites for several important molecules such as PI3K which could phosphorylate ERK, indicating that MUC1 could participate in the ERK phosphorylation signaling [52–54]. In the future, we will further explore the molecular mechanism by which MUC1 regulates ERK phosphorylation in cervical squamous cell carcinoma.

To further clarify the molecular mechanism of MUC1 in CSCC, we screened the genes whose expression was downregulated in the related GSEA sets and found that the expression of ITGAs decreased after MUC1 knockout. The integrin α (ITGA) subfamily of genes plays a fundamental role in various cancers [55]. ITGA2 was significantly upregulated in high-grade serous ovarian cancers compared with normal counterparts, and overexpressed ITGA2 contributed to paclitaxel resistance via activation of the AKT pathway in ovarian cancer cells [56, 57]. ITGA3 also serves as a favorable prognostic biomarker and contributes to several types of cancers, such as breast cancer [58, 59]. However, the role of ITGAs in cervical squamous cell carcinoma has not yet been studied. In the present study, we found that the mRNA level of ITGA2 and ITGA3 were increased in cervical cancer patients via the TCGA database. The expression of MUC1 was stable after ITGA2 and ITGA3 were reduced, indicating that ITGA2 and ITGA3 are the downstream of MUC1. We treated cervical cancer cells with a new inhibitor of ERK (MK-8353) and found that the expression of ITGA2 and ITGA3 was significantly reduced. These findings suggested that the regulation of ITGA2 and ITGA3 was achieved through MUC1-mediated ERK phosphorylation. Furthermore, we explored the role of ITGA2 and ITGA3 in promoting tumorigenesis in CSCC. ITGA2 and ITGA3 knockdown inhibited the tumorigenic phenotype in CSCC.

The overexpression of MUC1 contributes to the drug resistance in several cancer types [60]. High MUC1 expression has been reported to limit the effectiveness of antitumor drugs by reducing intracellular drug uptake in pancreatic tumors [61]. MUC1 can also stabilize the expression of HIF1A and increase glycolytic flux in cancer cells to reduce the sensitivity of tumor tissue to anti-tumor drugs [62]. Therefore, inhibiting the expression of MUC1 is highly important for tumor treatment. Here, we found that abnormal MUC1 expression could increase the phosphorylation level of ERK. Notably, when MUC1 was knocked out, the effect of MK-8353, a novel ERK inhibitor, was also inhibited. Based on these findings, we designed a therapeutic strategy in which MUC1 and downstream signaling pathways were inhibited. We used MK-8353 to inhibit ERK phosphorylation in combination with in vivo transfection of siRNA targeting MUC1 and found that the combination regimen achieved better outcomes in animals with CSCC xenografts than did MUC1 alone. These results suggest that targeting MUC1 combined with inhibition of the downstream ERK phosphorylation may be a more useful method for treating patients with high MUC1 expression in future clinical trials.

Taken together, these findings reveal a novel MUC1/ERK/ITGA_2/3_ regulatory pathway based on in vitro and in vivo assays. Our results suggest that this pathway may not only provide potential molecular markers for the early diagnosis of cervical cancer but may also serve as a new candidate target for cervical cancer therapy. However, the molecular mechanism of the ITGA family in promoting cervical cancer is still unclear and require further study. And we will extend a cohort with large number of cases to evaluate the value of MUC1 in predicting the occurrence of cervical squamous cell carcinoma in future studies.

### Electronic supplementary material

Below is the link to the electronic supplementary material.

Supplementary Material 1

Supplementary Material 2

Supplementary Material 3

Supplementary Material 4

## Data Availability

The datasets generated and analysed during the current study are available in the GEO repository [GSE241017] and the datasets (TCGA-CESC) analysed during the current study are available in the UCSC Xena TCGA repository (https://tcga.xenahubs.net).
