# Supplementary material for: MUC1 promotes cervical squamous cell carcinoma through ERK phosphorylation-mediated regulation of ITGA2/ITGA3
Source: BMC Cancer. 2024 May 3;24:559. doi: 10.1186/s12885-024-12314-6 (PMC11069143; doi:10.1186/s12885-024-12314-6)
Supplement: Supplementary file 3 — Supplementary Material 3 [file 12885_2024_12314_MOESM3_ESM.pdf]

MUC1 promotes cervical squamous cell carcinoma through ERK phosphorylation-mediated regulation of ITGA2/ITGA3

Aiqin Zhao[1,*], Yunzhi Pan[2,*], Yingyin Gao[3,4,*], Zheng Zhi[5], Haiying Lu[1], Bei Dong[1], Xuan Zhang[1], Meiying Wu[6], Fenxia Zhu[3,4], Sufang Zhou[1,#], Sai Ma[7,8,#]

1. Department of Obstetrics and Gynecology, The People's Hospital of Suzhou New District, Suzhou 215129, China
2. Department of Pharmacy, The Affiliated Infectious Diseases Hospital of Soochow University, Suzhou 215131, China
3. Affiliated Hospital of Integrated Traditional Chinese and Western Medicine, Nanjing University of Traditional Chinese Medicine, Nanjing, China. 210023
4. Key Laboratory of New Drug Delivery Systems of Chinese Materia Medica, Jiangsu Province Academy of Traditional Chinese Medicine, Nanjing 210028, China.
5. Department of Pathology, The Affiliated Suzhou Hospital of Nanjing Medical University, Suzhou 215002, China
6. Department of Tuberculosis, The Affiliated Infectious Diseases Hospital of Soochow University, Suzhou, 215131, China.
7. Department of Laboratory, The Affiliated Suzhou Hospital of Nanjing Medical University, Suzhou 215002, China
8. Gusu School, Nanjing Medical University, Suzhou, China. 215008

*, These authors contributed equally to this work.

#, **Correspondence authors:**

Sai Ma, Ph.D.

Department of laboratory, The Affiliated Suzhou Hospital of Nanjing Medical University, Gusu School, Nanjing Medical University, Suzhou, China. 215008

E-mail: marseillems@njmu.edu.cn. Tel & Fax: 0512-62363800

Sufang Zhou, MD.

Department of Obstetrics and Gynecology, The People`s Hospital of SND, Suzhou 2 215129, China. E-mail: zhousuf@sina.com. Tel & Fax: 0512-66612006

3A

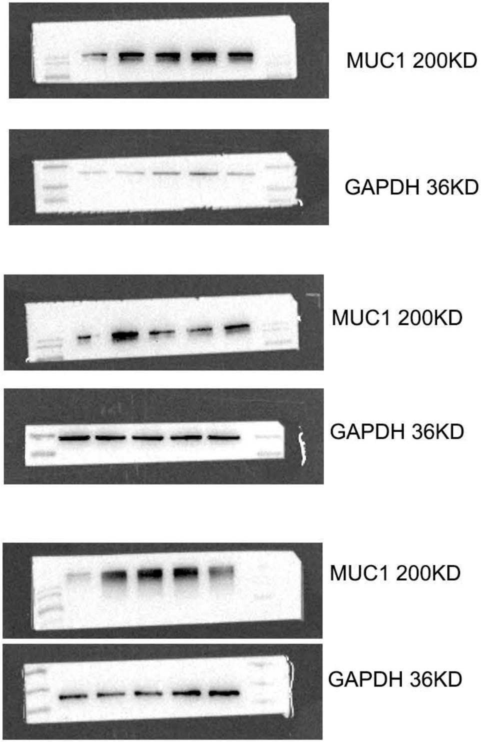
MUC1 200KD
GAPDH 36KD
MUC1 200KD
GAPDH 36KD
MUC1 200KD
GAPDH 36KD

3B

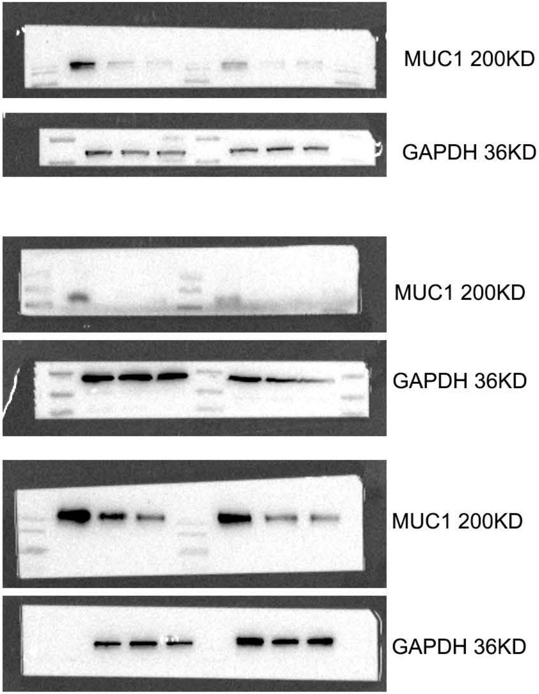
MUC1 200KD
GAPDH 36KD
MUC1 200KD
GAPDH 36KD
MUC1 200KD
GAPDH 36KD

4E

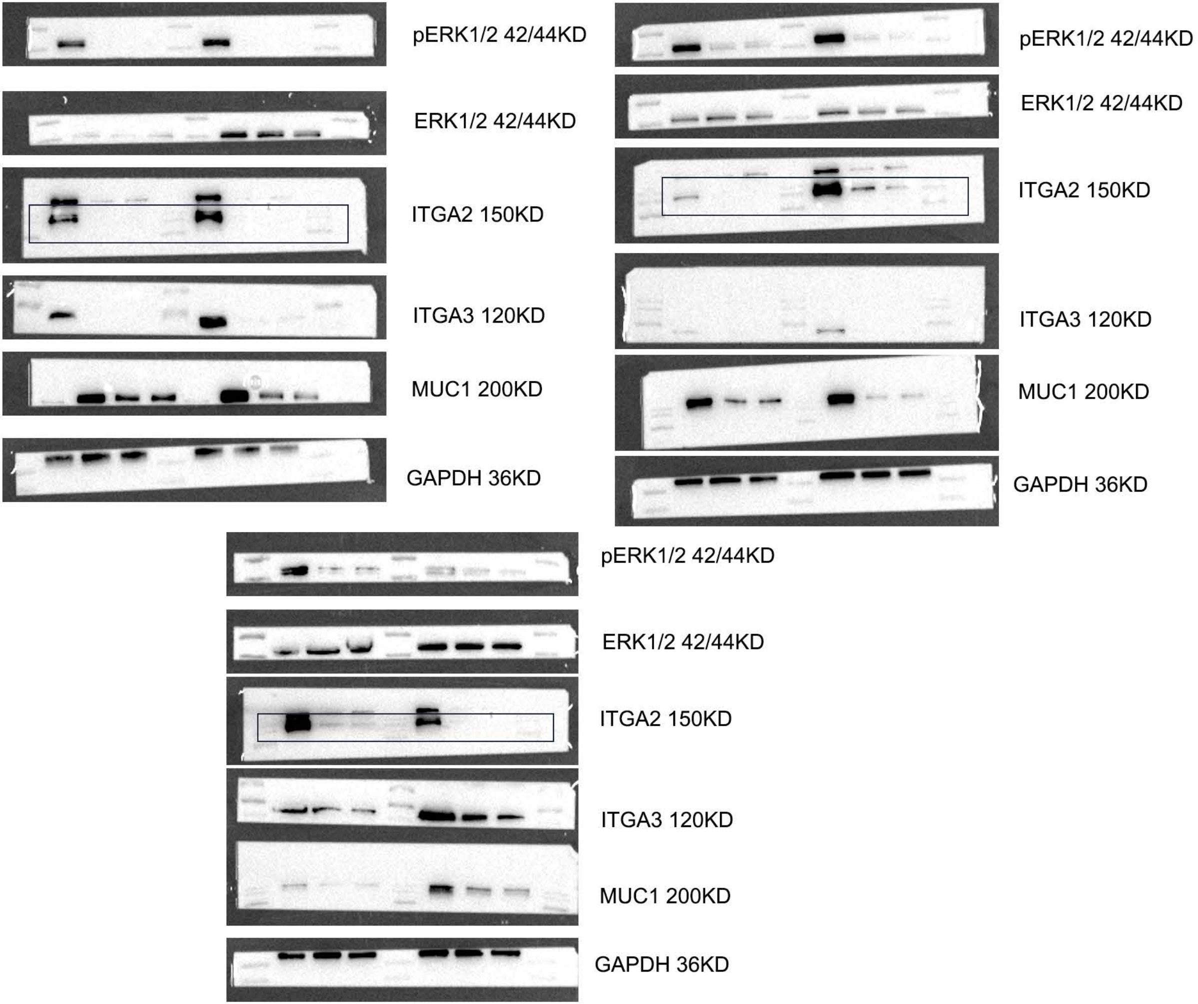
pERK1/2 42/44KD
ERK1/2 42/44KD
ITGA2 150KD
ITGA3 120KD
MUC1 200KD
GAPDH 36KD
pERK1/2 42/44KD
ERK1/2 42/44KD
ITGA2 150KD
ITGA3 120KD
MUC1 200KD
GAPDH 36KD
pERK1/2 42/44KD
ERK1/2 42/44KD
ITGA2 150KD
ITGA3 120KD
MUC1 200KD
GAPDH 36KD

4F

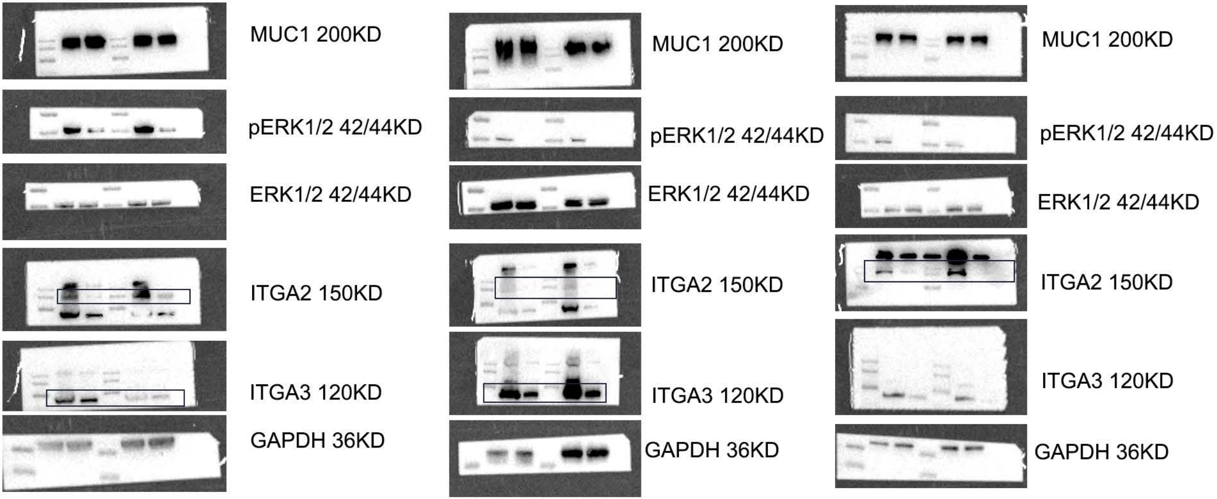
MUC1 200KD
pERK1/2 42/44KD
ERK1/2 42/44KD
ITGA2 150KD
ITGA3 120KD
GAPDH 36KD
MUC1 200KD
pERK1/2 42/44KD
ERK1/2 42/44KD
ITGA2 150KD
ITGA3 120KD
GAPDH 36KD
MUC1 200KD
pERK1/2 42/44KD
ERK1/2 42/44KD
ITGA2 150KD
ITGA3 120KD
GAPDH 36KD

5A

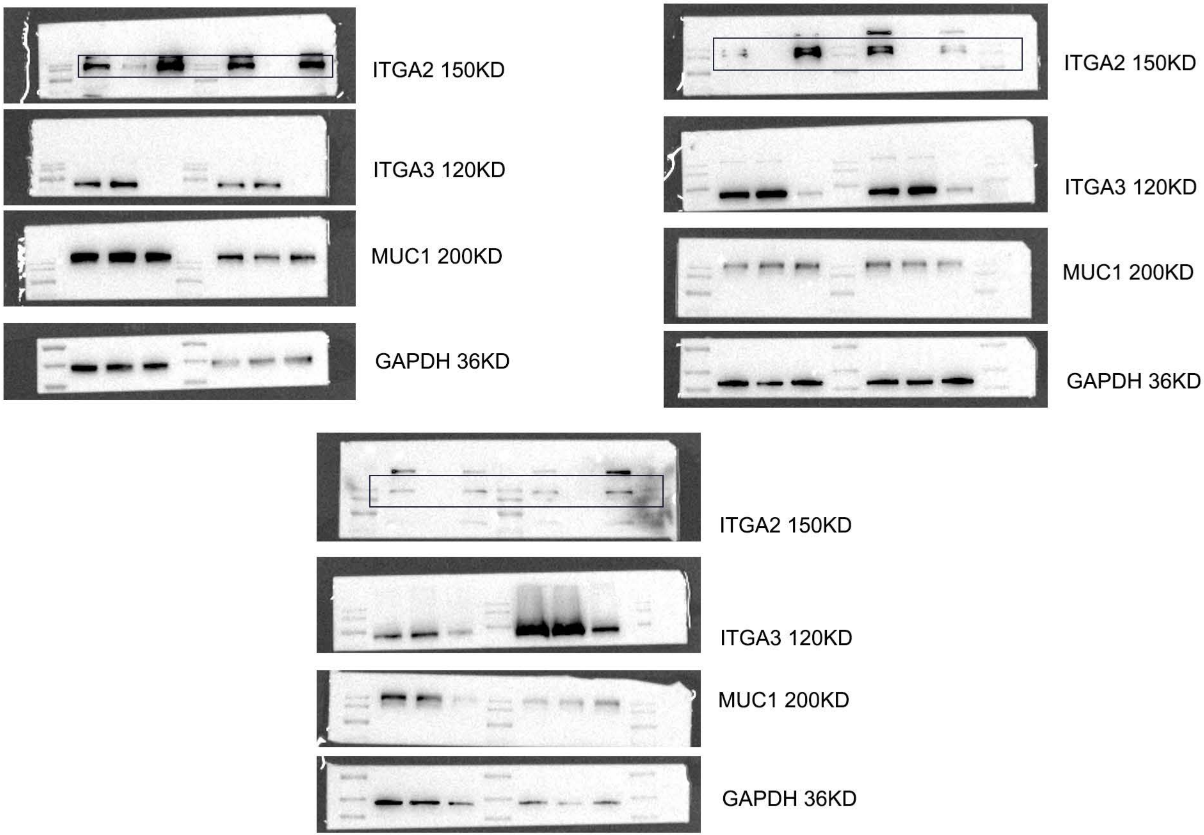
ITGA2 150KD
ITGA3 120KD
MUC1 200KD
GAPDH 36KD
ITGA2 150KD
ITGA3 120KD
MUC1 200KD
GAPDH 36KD
ITGA2 150KD
ITGA3 120KD
MUC1 200KD
GAPDH 36KD
