# Supplementary material for: MUC1 promotes cervical squamous cell carcinoma through ERK phosphorylation-mediated regulation of ITGA2/ITGA3
Source: BMC Cancer. 2024 May 3;24:559. doi: 10.1186/s12885-024-12314-6 (PMC11069143; doi:10.1186/s12885-024-12314-6)
Supplement: Supplementary file 4 — Supplementary Material 4 [file 12885_2024_12314_MOESM4_ESM.docx]

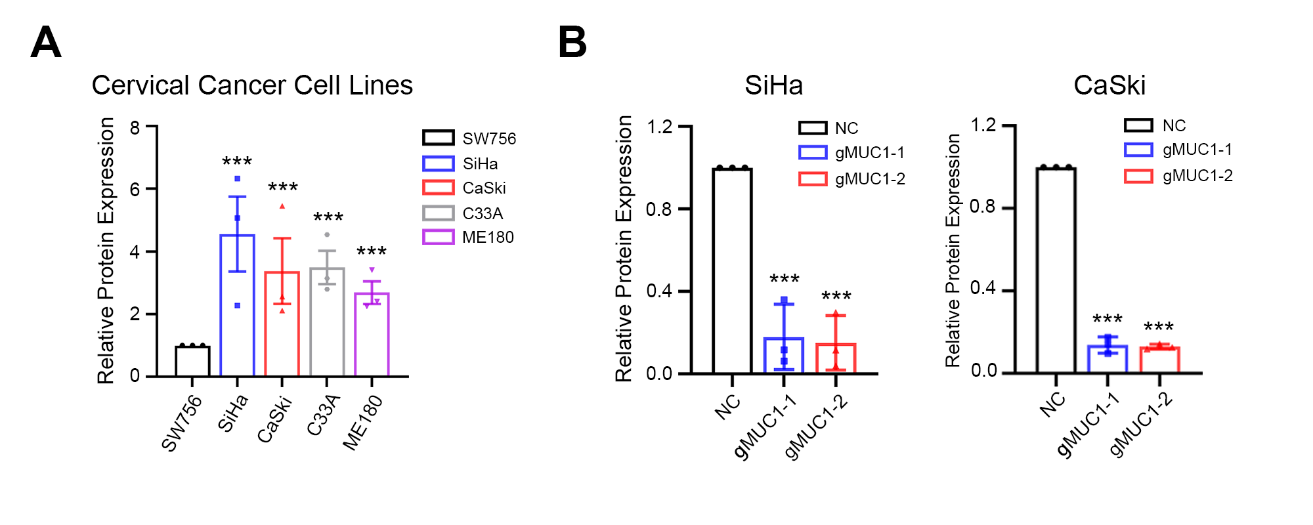


Supplementary Figure 1

1. Quantitation protein expression of MUC1 relative to GAPDH in cervical squamous cell carcinoma cell lines, n=3.
2. Quantitation protein expression of MUC1 relative to GAPDH in SiHa and CaSki cells after sgRNA transfection, n=3.

The means ± SEM are shown. ***, p<0.001; n.s., not significant.


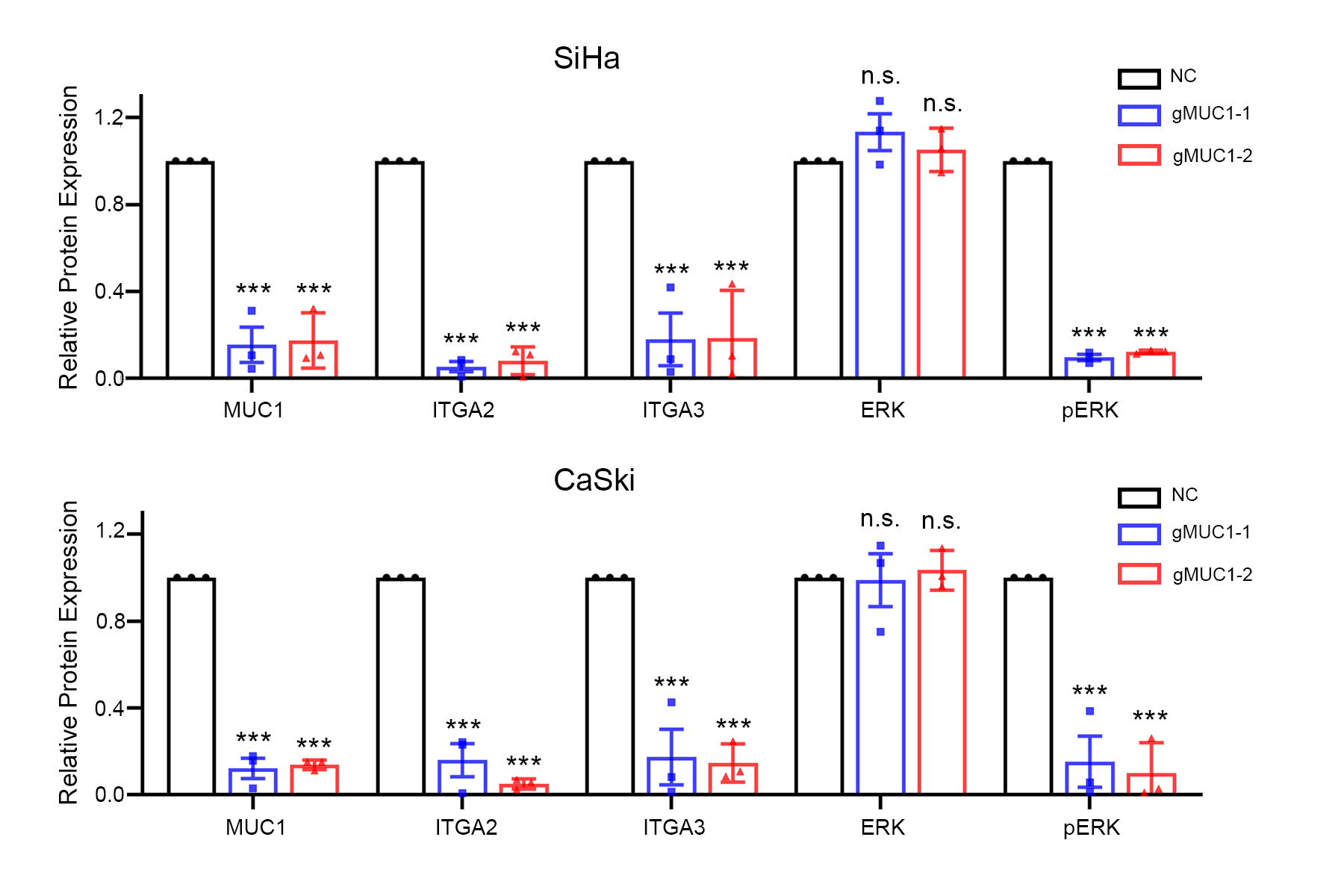


Supplementary Figure 2

Quantitation protein expression of p-ERK, ERK, ITGA2, ITGA3 and MUC1 relative to GAPDH in negative control and MUC1 knockout SiHa and CaSki cells, n=3. The means ± SEM are shown. ***, p<0.001; n.s., not significant.


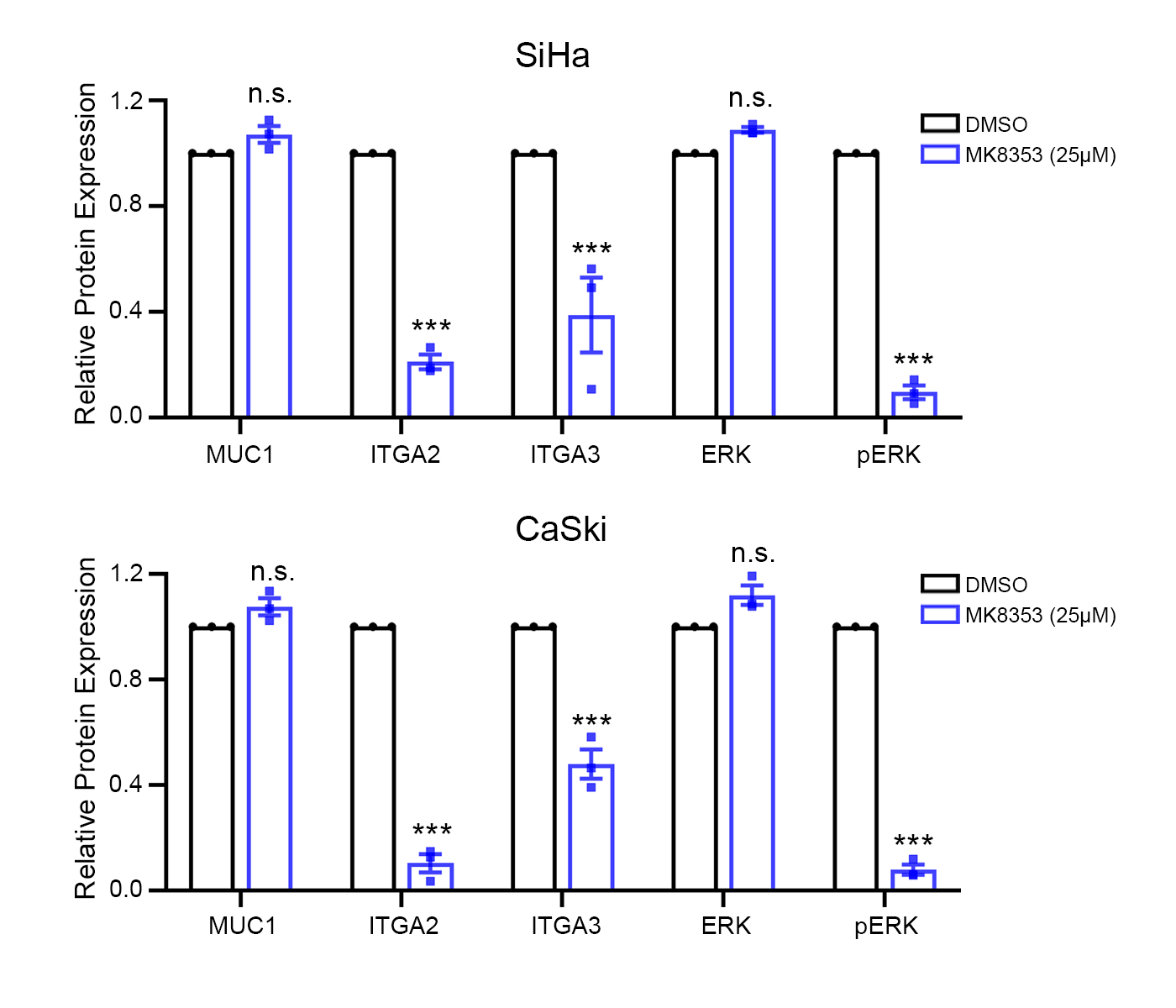


Supplementary Figure 3

Quantitation protein expression of MUC1, p-ERK, ERK, ITGA2 and ITGA3 relative to GAPDH in SiHa and CaSki cells treated with DMSO or the ERK inhibitor MK-8353, n=3. The means ± SEM are shown. ***, p<0.001; n.s., not significant.


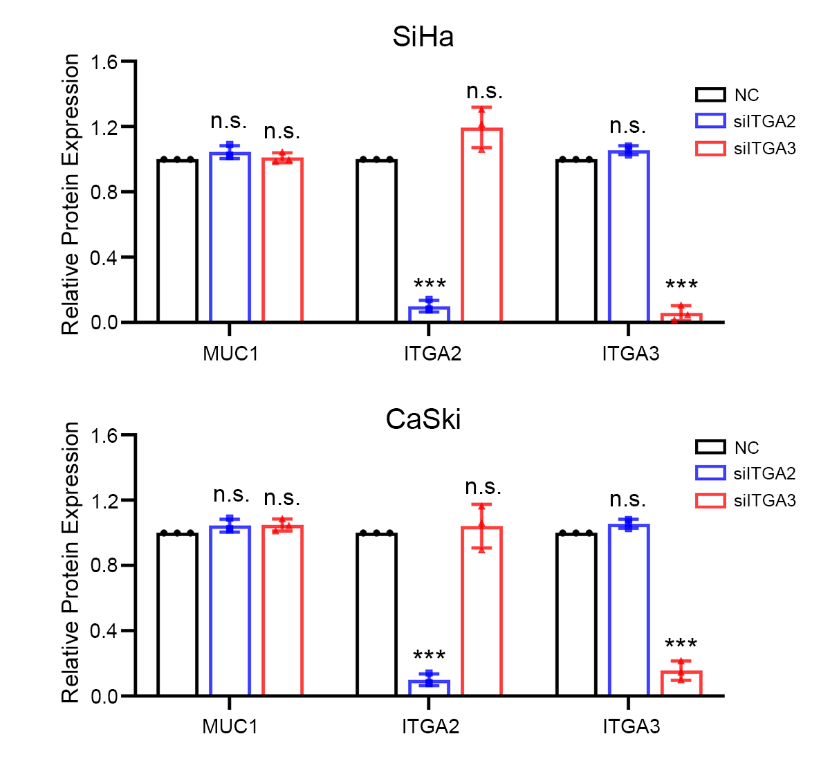


Supplementary Figure 4

Quantitation protein expression of ITGA2, ITGA3 and MUC1 relative to GAPDH in SiHa and CaSki cells after ITGA2 or ITGA3 knockdown, n=3. The means ± SEM are shown. ***, p<0.001; n.s., not significant.


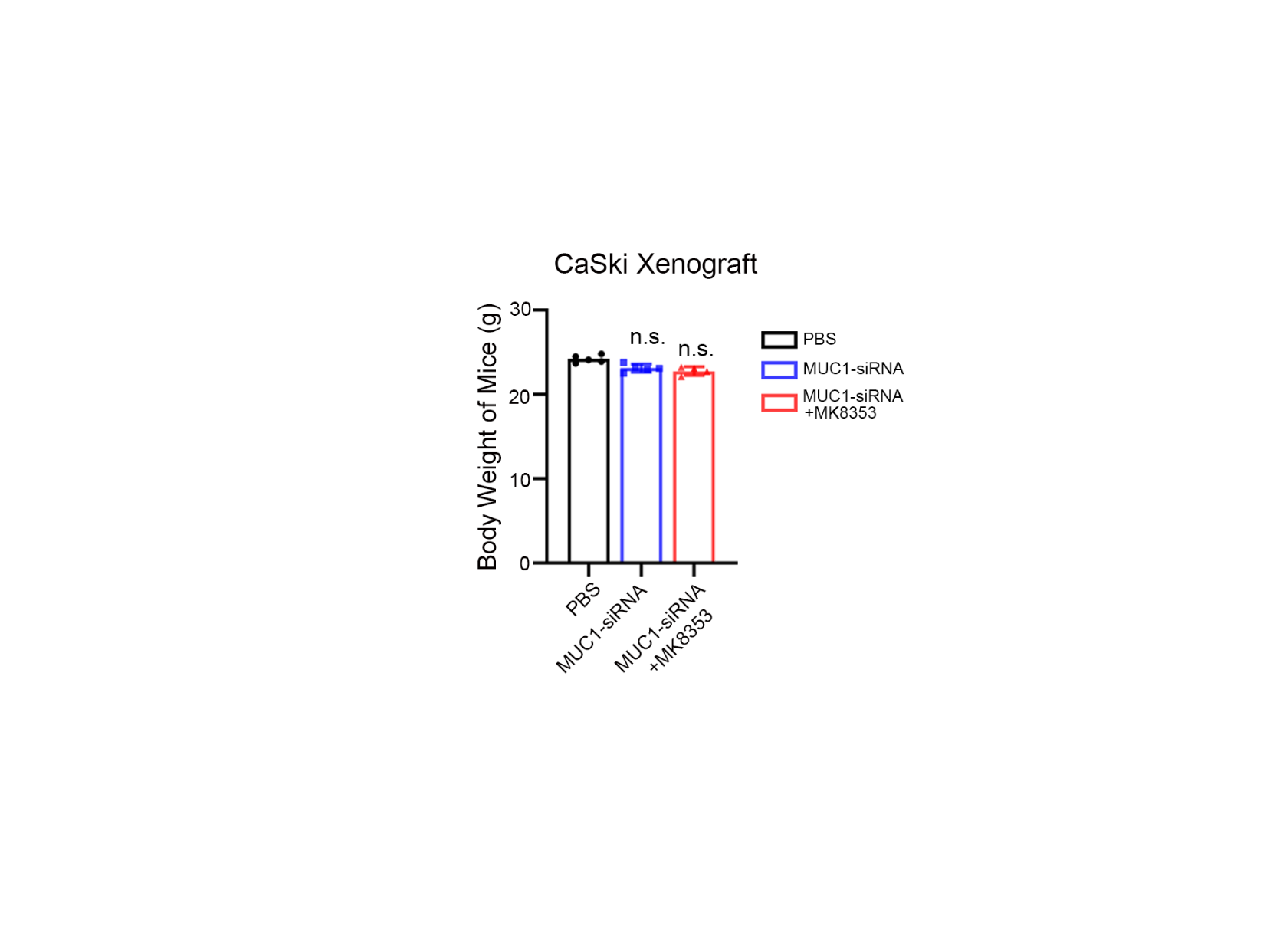


Supplementary Figure 5

The body weight of mouse from the indicated groups, n=5. The means ± SEM are shown. n.s., not significant.
